# Neoadjuvant Therapy Using Checkpoint Inhibitors before Radical Cystectomy for Muscle Invasive Bladder Cancer: A Systematic Review

**DOI:** 10.3390/jpm11111195

**Published:** 2021-11-13

**Authors:** Hadi SHSM, Usama A. Fahmy, Nabil A. Alhakamy, Mohd G. Khairul-Asri, Omar Fahmy

**Affiliations:** 1 Department of Urology, Royal Cornwall Hospital, Truro TR1 3LJ, UK; hadi.mohsin@nhs.net; 2 Department of Pharmaceutics & Industrial Pharmacy, Faculty of Pharmacy, King Abdulaziz University, Jeddah 21589, Saudi Arabia; uahmedkauedu.sa@kau.edu.sa; 3 Advanced Drug Delivery Research Group, Faculty of Pharmacy, King Abdulaziz University, Jeddah 21589, Saudi Arabia; nalhakamy@kau.edu.sa; 4 Center of Excellence for Drug Research and Pharmaceutical Industries, King Abdulaziz University, Jedah 21589, Saudi Arabia; 5 Department of Urology, Universiti Putra Malaysia (UPM), Serdang 43400, Selangor, Malaysia; khairulasri@upm.edu.my

**Keywords:** bladder cancer, immunotherapy, checkpoint inhibitors, radical cystectomy, chemotherapy

## Abstract

Background: Neoadjuvant chemotherapy is the standard of care before radical cystectomy for muscle invasive bladder cancer. Recently, checkpoint inhibitors have been investigated as a neoadjuvant treatment after the reported efficacy of checkpoint inhibitors in metastatic urothelial carcinoma. Objectives: The aim of this systematic review is to investigate the role of checkpoint inhibitors as a neoadjuvant treatment for muscle invasive bladder cancer before radical cystectomy. Methods: Based on the PRISMA statement, a systematic review of the literature was conducted through online databases and the American Society of Clinical Oncology (ASCO) Meeting Library. Suitable publications were subjected to full-text assessment. The primary outcome of this review was to identify the impact of neoadjuvant immunotherapy on the oncological outcomes and survival benefits. Results: From the retrieved 254 results, 8 studies including 404 patients were included. Complete response varied between 30% and 50%. Downstaging varied between 50% and 74%. ≥Grade 3 AEs were recorded in 8.6% of patients who received monotherapy with either Atezolizumab or Pembrolizumab. In patients who received combination treatment, the incidence of ≥Grade 3 AEs was 16.3% for chemoimmunotherapy and 36.5% for combined immunotherapy. A total of 373 patients (92%) underwent radical cystectomy. ≥Grade 3 Clavien-Dindo surgical complications were reported in 21.7% of the patients. One-year overall survival (OS) and relapse-free survival (RFS) varied between 81% and 92%, and 70% and 88%, respectively. Conclusion: The evidence on the use of immune checkpoint inhibitors in the setting of pre-radical cystectomy is quite limited, with noted variability within published trials. Combination with chemotherapy or another checkpoint inhibitor may boost response, although prospective studies with extended follow-up are needed to report on the survival advantages.

## 1. Introduction

Muscle invasive bladder cancer remains one of the most lethal forms of urothelial carcinoma. Untreated, the 5-year overall survival rate is less than 5% [1]. Despite the advancements in peri-operative treatment regimens including neoadjuvant and adjuvant treatment, coupled with timely radical cystectomy (RC) and lymph node dissection, a significant proportion of patients eventually develop metastatic disease [2].

The use of immune mediated treatment in non-muscle invasive bladder cancer has been well documented with the widespread use of Bacillus Calmette–Guérin (BCG) instillation, BCG treatment, first introduced by Morales in 1976 [3]. Via intravesical instillation of BCG, infiltration of cytotoxic T lymphocytes (CTLs) and cell-mediated cytotoxicity against bladder tumors occurs through activation of innate and adaptive immunity [4]. This formed the basis for immunological manipulation of the bladder mucosa in preventing the recurrence of high-risk non-muscle invasive urothelial carcinoma.

The use of platinum-based chemotherapy as a neoadjuvant treatment prior to cystectomy is well established and common practice [5]. The combination of gemcitabine and cisplatin is the standard first line therapy in the metastatic setting with improvement in median survival of up to 14 months compared to just 6 months [6]. Indeed, numerous trials have elucidated the significant overall survival benefit of the use of neoadjuvant platinum-based chemotherapy in invasive urothelial carcinoma [7].

Recent developments with immune checkpoint inhibitors (ICIs) have shown efficacy in locally advanced and metastatic urothelial carcinoma [8]. A few years ago, the Food and Drug Administration (FDA) approved five immune checkpoint inhibitors (ICIs) for use in various contexts, including first line and second-line therapy for metastatic urothelial carcinoma. Pembrolizumab, a programmed death−1 (PD−1) inhibitor, was recently licensed for high-risk Bacillus Calmette‒Guérin (BCG)-unresponsive non-muscle invasive bladder cancer (NMIBC) employing immunotherapy at an early stage of the illness [9]. Very recently, Nivolumab was approved as an adjuvant treatment for high-risk muscle invasive urothelial carcinoma, after showing a better disease-free survival rate compared to the placebo [10].

Whereas numerous trials in the perioperative setting are currently continuing, the role of immune checkpoint inhibition in the neoadjuvant setting is still not clear. This review aims to systematically appraise the emerging role of immune checkpoint inhibition in the neoadjuvant setting for muscle invasive urothelial carcinoma in terms of efficacy and tolerability.

## 2. Materials and Methods

### 2.1. Search Strategy

Based on the PRISMA statement [11], a systematic online search was conducted through online databases (PubMed, EMBASE, Web of Science, Wiley online library and Cochrane databases), in addition to the American Society of Clinical Oncology (ASCO) Meeting Library. The following keywords were used: bladder cancer, radical cystectomy, neoadjuvant, chemotherapy, immunotherapy, checkpoint inhibitors, anti-CTLA−4, and anti-PD-L. An initial assessment of titles and abstracts of all retrieved results was performed with subsequent exclusion of the unrelated articles, case reports, editorials, and review articles. Eligible publications were subjected to full-text assessment followed by exclusion of duplications and other articles unrelated directly to the topic. In addition, a manual search was performed in the references list of the selected papers to avoid missing any eligible publication.

### 2.2. Data Extraction

The following variables were extracted: total number of patients, number of the patients who underwent RC, gender, age, follow-up duration, type of neoadjuvant treatment, adverse events (AEs), RC complications, and survival data. For quantitative analysis, the number of events and total number of each subgroup were extracted. Data were extracted independently by two authors, then a double-check was performed for accuracy.

### 2.3. Primary Outcomes

The primary outcome of this analysis was the impact of neoadjuvant immunotherapy on the outcome of radical cystectomy, in terms of complete response (CR), downstaging (DS), tolerability and AEs, survival benefits, and factors associated with higher response. CR was defined as pT0, meanwhile, DS was defined as ≤pT1.

### 2.4. Statistical Analysis

The Nordic Cochrane Centre, The Cochrane Collaboration, Copenhagen, employed Review Manager (RevMan) software version 5.4 for statistical analysis and the creation of forest plots for the quantitative analysis and calculation of the odds ratio (OR) and 95% confidence interval (CI). The *I*^2^ value was used to determine the heterogeneity of the research. For *I*^2^ < 50%, a fixed effect model was used, but for *I*^2^ ≥ 50%, a random effect model was examined. The Z-test was used to assess the overall impact. A *p*-value <0.05 was employed as the significance level.

### 2.5. Risk of Bias Assessment

The Newcastle-Ottawa Scale (NOS) was employed in this meta-analysis to assess the quality of non-randomized trials [12]. Scores of 7–9, 4–6, and less than 4 were classified as having a low, moderate, or high risk of bias, respectively.

## 3. Results

### 3.1. Search Results

**Figure 1:**
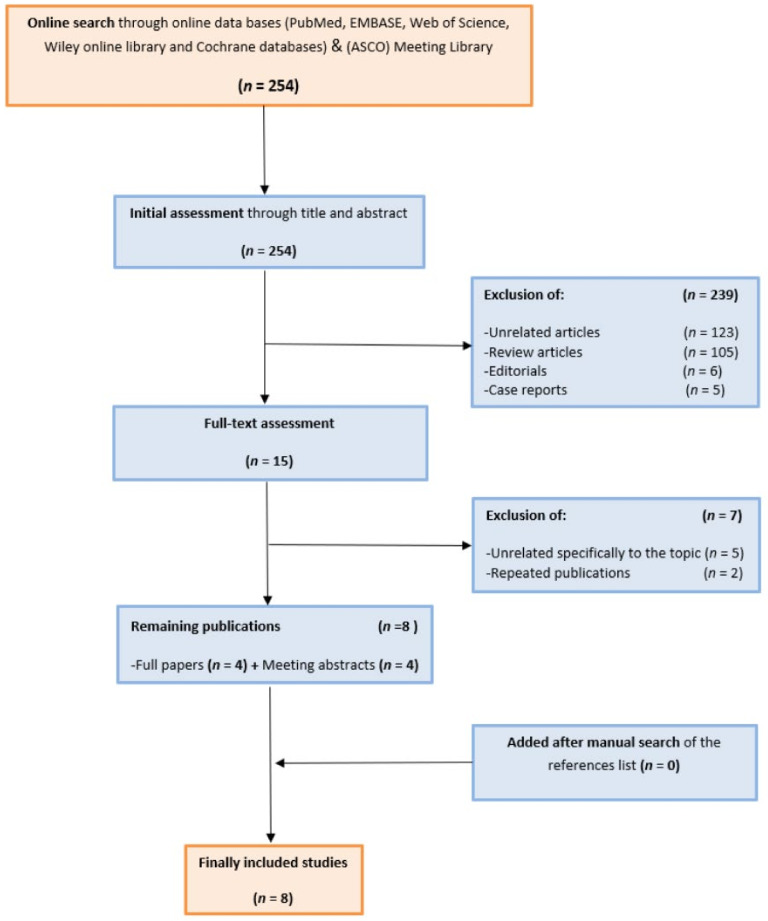
CONSORT diagram for the screening and selection processes of the included studies.

An initial search retrieved 254 results, which underwent assessment to identify the eligible publications. Finally, 8 studies (4 papers and 4 abstracts), including 404 patients were included [13,14,15,16,17,18,19,20]. The screening and selection processes are demonstrated in Figure 1. Different protocols were used in those studies; Atezolizumab (MPDL3280 A) was only investigated as a monotherapy [13]. Pembrolizumab was investigated as a monotherapy [14], or in combination with chemotherapy [18]. Three studies investigated Durvalumab in combination with other drugs, with chemotherapy in two studies [15,16] and with Termelimumab (anti-CTLA−4) in the one study [20]. Nivolumab was investigated in two studies, in combination with chemotherapy [17] and in combination with Ipilimumab (anti-CTLA−4) [19]. A summary of the studies and patients’ criteria is presented in Table 1. Based on NOS bias assessment, all the included studies had moderate risk of bias. (Table 2).

### 3.2. Oncological Response

Reported CR varied between 30% and 50% among the studies [15,16]. CR in monotherapy was slightly lower compared to combination therapy. In 209 patients treated with either Atezolizumab or Pembrolizumab, the average CR was 34% [13,14]. In 103 patients from three studies, combination of immunotherapy and chemotherapy displayed CR in 40% [15,16,18]. Furthermore, combined immunotherapy via combination of PD−1 drugs or PD-L1 with anti-CTLA−4 showed CR in 40% in 52 patients from two studies [19,20].

**Figure 2:**
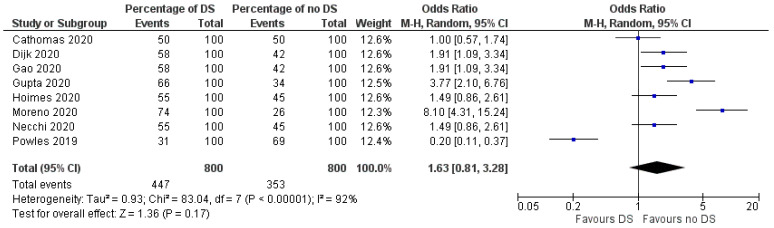
Forest plot for the quantitative analysis of odds ratio for possibility of downstaging (DS).

DS varied between 50% and 74% [15,16]. In 114 patients treated with Pembrolizumab, DS was 55% [14]. In four studies investigating combined immuno-chemotherapy, 60% of 144 patients showed DS [15,16,17,18], and in a combination of PD−1 agents or PD-L1 with anti-CTLA−4, DS was 58% in 52 patients [19,20]. Quantitative analysis of the possibility of DS including all studies displayed an OR of 1.63 (0.81–3.28) (Figure 2).

### 3.3. Safety and Side Effects

**Figure 3:**
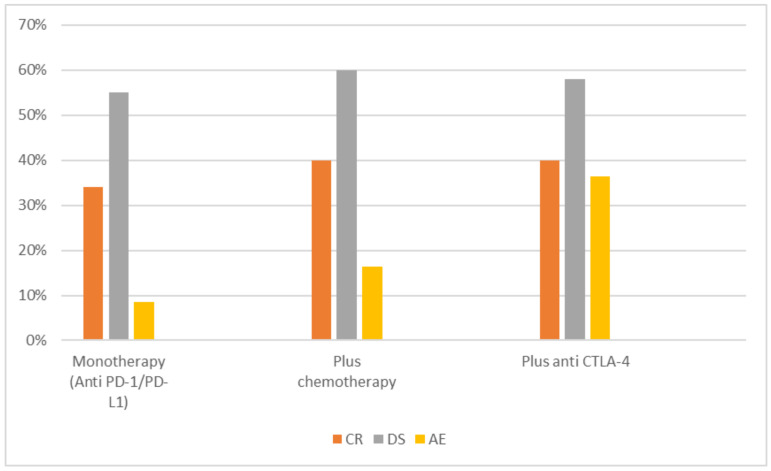
Comparison of the incidence of complete response (CR), downstaging (DS) and adverse events (AE) between anti-PD−1/PD-L1 as monotherapy or in combination with chemotherapy or anti-CTLA−4.

There was a variation in reporting the AEs. Most of the studies reported on serious AEs only (≥Grade 3) [15,16,17,19]. Three studies reported on all AEs [13,14,20]. ≥Grade 3 AEs varied between 3% and 55% [15,19]. In patients who received monotherapy with either Atezolizumab or Pembrolizumab, ≥Grade 3 AEs were recorded in 8.6% [13,14]. Meanwhile, combination therapy was reported to have higher ≥Grade 3 AEs. In 104 patients who received combined immune and chemotherapy, the incidence of ≥Grade 3 AEs was 16.3% [15,16,17], and in 52 patients who received combined PD−1 or PD-L1 with anti-CTLA−4, it was 36.5% [19,20]. Figure 3 displays the comparison between mono and combined therapy in outcome and ≥Grade 3 AEs.

### 3.4. Surgical Complications

Of the 404 patients included in this review, 373 (92%) underwent radical cystectomy. Surgical complications were reported in five studies [13,14,15,16,20]. Overall incidence of surgical complications was 56% (142/253 patients) [13,14,16,20]. ≥Grade 3 Clavien-Dindo surgical complications were reported in 21.7% (54/249 patients) [13,14,15,16,21].

### 3.5. Survival Outcomes

There are no available data on long-term survival outcomes. Only 1-year overall survival (OS) and relapse free survival (RFS) were reported in 4 studies, and varied between 81% and 92%, and 70% and 88%, respectively [13,18,19,20]. A summary of the outcome of the included studies is provided in Table 3.

### 3.6. Subgroup Quantitative Analysis

**Figure 4:**
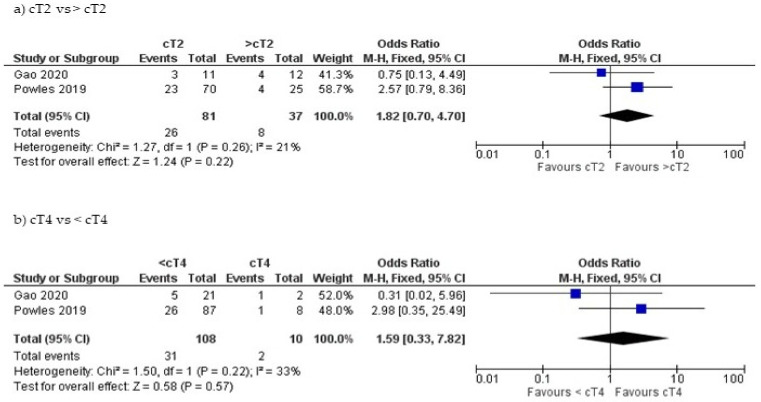
Forest plot for the impact of clinical stage of bladder cancer on complete response rate. (**a**) cT2 vs. > cT2, (**b**) cT4 vs. < cT4.

**Figure 5:**
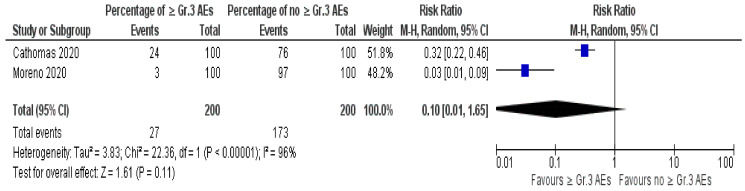
Forest plot of the risk ratio of developing ≥ Grade 3 adverse events due to combination of Durvalumab and chemotherapy.

Subgroup quantitative analysis was feasible to assess the CR based on the clinical stage and PD-L1 positivity. In comparing patients with cT2 disease and patients with cT3 or cT4, CR was achieved in 32% (26/81) and 21.6% (8/37), respectively. However, the difference was not significant (OR = 1.82; Z = 1.24; *p* = 0.22) (Figure 4a). When cT4 patients were compared to cT2 or cT3, the CR rate was 20% (2/10) for cT4 vs 28.7% (31/108) (OR = 1.59; Z = 0.58; *p* = 0.57) [13,20] (Figure 4b). The risk ratio (RR) of developing ≥ Grade 3 AEs due to combination of Durvalumab and chemotherapy was calculated from the two studies using the same combination and showed a RR of 0.10 (0.01–1.65) [15,16] (Figure 5).

**Figure 6:**
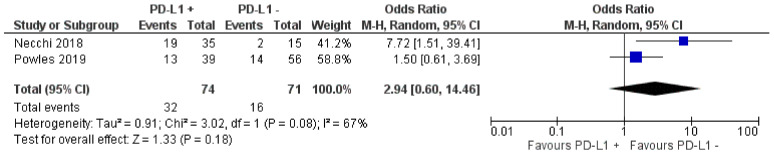
Forest plot for the impact of PD-L1 positivity on complete response rate.

Investigation of CR based on PD-L1 positivity displayed a trend towards higher CR in PD-L1 positive patients, yet it did not reach the significance level. CR was 43.2% (32/74) in positive patients vs 22.5% (16/71) in negative patients. (OR = 2.94; Z = 1.33; *p* = 0.18) (Figure 6) [13,22].

NB: Necchi 2018 [22] is the early report for the PURE-01 trial as the data included in this forest plot was not available in Necchi 2020 [14].

## 4. Discussion

It has been reported that in the setting of urothelial carcinoma, advanced and aggressive tumors with poor survival outcomes are seen in cancers which express high levels of programmed death-ligand 1 (PD-L1) expression [23]. It is postulated that the antitumor immune response is improved by ICIs due to a greater T cell-mediated antitumor immune response which is elicited by the greater availability of neoantigens [24].

Atezolizumab became the first new drug approved in metastatic urothelial carcinoma in over 30 years followed by nivolumab in 2017 [25]. This has sparked interest in the possible role of immune checkpoint inhibitors in the neoadjuvant setting. Current trials find a role for immune checkpoint inhibitors in the neoadjuvant setting for patients who are contraindicated for platinum-based therapy mainly due to renal impairment [25].

Unlike standard neoadjuvant combination chemotherapy, the role of neoadjuvant PD-L1 inhibitors in muscle invasive bladder cancer is not well established. Some preliminary studies have looked at the role of PD-L1 inhibitors as a neoadjuvant treatment, in a select group of patients who are unfit or did not respond to chemotherapy [14].

In our systematic review, we looked at the role of PD-L1 inhibitors in the neoadjuvant setting. Two broad categories were identified; the use of PD-L1 inhibitors as monotherapy or in combination with either CTL-4 inhibitors or chemotherapy.

In terms of survival, we observed that combination chemotherapy and PD-L1 inhibitors showed only a modest improvement in complete response compared to PD-L1 inhibitor monotherapy or combination therapy. This may call into question the rationale of combination therapy, with its attendant higher risk of toxicity and also costs in this setting.

The downstaging was comparable between all three categories of patients: having either PD-L1 inhibitor monotherapy. In combination with chemotherapy or combination with a CTL−4, downstaging was variable across all groups.

Regardless of this variable response, the results are promising, especially for the subset of patients who may not be suitable for neoadjuvant chemotherapy. The role of neoadjuvant treatment in the setting of muscle invasive bladder cancer is well established and proven to reduce relapse and improve survival by eliminating micro-metastatic deposits prior to radical cystectomy [26]. In all the studies reviewed, an average of 90% of patients went on to have a radical cystectomy.

An interesting observation was seen in the analysis of safety and adverse events. Inhibition of autoimmunity and limitation of immune activation which occurs with administration of these medications are thought to contribute to a wide range of side effects resembling autoimmune reactions. The rationale for dual immune checkpoint inhibition lies in its potential for synergistic immunotherapy activity and thus efficacy [27].

Whilst it was not surprising to see a higher reported rate of side effects and surgical complications in the combination of chemotherapy and PD-L1 versus PD-L1 monotherapy, the studies looking at combination of PD-L1 and CTL−4 consistently reported a higher complication rate.

In terms of survival, only four studies looked into the analysis of recurrence-free survival and overall survival. As the role of immune checkpoint inhibition in the neoadjuvant setting of muscle invasive bladder cancer is still in its infancy, this is not surprising as insufficient time has lapsed to allow results to accrue. Nevertheless, RFS in the region above 70% and OS of over 80% across all studies look to be very promising. Furthermore, based on a recent systematic review and meta-analysis by Fahmy et al., complete downstaging to T0 before radical cystectomy is associated with better survival outcomes [2]. Therefore, the observed CR and DS in this review could be reflected in better survival in the future.

This study is limited by the wide disparity between the included studies in the various treatment regiments and combinations of therapeutic agents. These might carry additional confounders for the observations seen. Furthermore, all the studies are phase I or II, with a small number of patients and lacking a control arm for comparison. All that limited the chance for meaningful quantitative analysis for pooled outcomes. In addition, all patients are from the USA or Europe, which might not be reflective of the global ethnical variations. However, from a practical point of view, adequate global participation can be limited by the availability of drugs and the difference between national health insurance systems among the countries. It will also be particularly interesting to see long-term survival data to allow more direct comparison between the use of immune checkpoint inhibition versus standard chemotherapy in this setting.

We have no doubt that as the role of immune checkpoint inhibition expands across all types of solid cancers and there is longer data accrual, its role in the neoadjuvant setting may become more established. Nevertheless, this review suggests the promising role it currently has in the expansion of our armamentarium in fighting muscle invasive bladder cancer.

## 5. Conclusions

The data on application of immune checkpoint inhibitors in the pre-radical cystectomy setting is very limited with heterogeneity observed among published studies. Combination with chemotherapy or other checkpoint inhibitors might improve the response; however, prospective trials with longer follow-up is required to report on the survival benefits. Identification of selection criteria for patients who can maximally benefit from this treatment modality ought to be aimed for in future trials.

## Figures and Tables

**Table 1:** Summary of the included studies.

Study	Country	Trial Name	Type	Neoadjuvant Treatment	No. of Patients	Male/Female	Mean Age
Powles 2019 [13]	UK	ABACUS	single-arm phase II	Atezolizumab	95	81/14	73
Necchi 2020 [14]	Italy	PURE−01	single-arm phase II	Pembrolizumab	114	99/15	66
Moreno 2020 [15]	Spain	NEODURVARIB	single-arm phase II	Durvalumab + Chemo	29	26/3	71
Cathomas 2020 [16]	Switzerland	SAKK−06/17	single-arm phase II	Durvalumab + Chemo	34	27/7	70
Gupta 2020 [17]	USA	BLASST−1	single-arm phase II	Nivolumab + Chemo	41	-	-
Hoimes 2020 [18]	USA	-	3-arm Phase Ib/II	Pembrolizumab + Chemo	40	30/10	65
Dijk 2020 [19]	Netherland	NABUCCO	single-arm phase I	Nivolumab + Ipilimumab	24	18/6	65(50–81)
Gao 2020 [20]	USA	-	single-arm phase I	Durvalumab + Termelimumab	27 *	20/8	71(24–83)

* Original sample size is 28 but we excluded one patient with primary T1 disease.

**Table 2:** Newcastle-Ottawa Scale for risk of bias assessment of the included studies (scores ≥7–9, 4–6, ˂4 are considered as low, intermediate, and high risk, respectively).

Study	Selection				Comparability	Outcome			Overall
	Representativeness of Exposed Cohort	Selection of Nonexposed	Ascertainment of Exposure	Outcome not Present at Start		Assessment of Outcome	Adequate Follow-up Length	Adequacy of Follow-up	
Powles 2019 [13]	*		*	*		*	*	*	6/9
Necchi 2020 [14]	*		*	*		*	*	*	6/9
Moreno 2020 [15]	*		*	*		*	*		5/9
Cathomas 2020 [16]	*		*	*		*	*		5/9
Gupta 2020 [17]	*		*	*		*	*		5/9
Hoimes 2020 [18]	*		*	*		*	*		5/9
Dijk 2020 [19]	*		*	*		*	*	*	6/9
Gao 2020 [20]	*		*	*		*	*	*	6/9

* One star for each item except the comparability is assessed by 2 stars (total score is 9 stars).

**Table 3:** Summary of the outcome of the included studies.

Study	DS	CR	AE (%)		RC (%)	Surgical Complications (%)		1 y OS	1 y RFS
			All Grades	≥ Grade 3		All Grades	≥ Grade 3		
Powles 2019 [13]	-	31%	49/95 (52)	10/95 (11)	87/95 (92)	54/87 (62)	15/87 (17)	-	79%
Necchi 2020 [14]	55%	37%	85/114 (75)	8/114 (7)	112/114 (98)	69/112 (62)	26/112 (23)	-	-
Moreno 2020 [15]	74%	50%	NA	1/29 (3)	26/29 (90)	NA	5/20 (25)	-	-
Cathomas 2020 [16]	50%	30%	NA	8/34 (24)	30/34 (88)	13/30 (43)	8/30 (27)	-	-
Gupta 2020 [17]	66%	-	NA	8/41 (20)	40/41 (98)	NA	NA	-	-
Hoimes 2020 [18]	55%	44%	NA	NA	36/40 (90)	NA	NA	94%	80%
Dijk 2020 [19]	58%	46%	NA	13/24 (55)	24/24 (100)	NA	NA	92%	88%
Gao 2020 [20]	58%	37%	25/27 (93)	6/27 (21)	23/27 (86)	5/23 (22)	NA	88%	82%

DS: downstaging; CR: complete response; AE: adverse events; RC: radical cystectomy; OS: overall survival; RFS: recurrence-free survival.

## Data Availability

Not applicable.
